# 

**Published:** 1907-01

**Authors:** 


SUBSCRIPTION $1.00.

PUBLISHED MONTHLY.

### ATLANTA JOURNAL-RECORD OF MEDICINE.

Successor to Atlanta Medical and Surgical Journal, Established 1855.
and Southern Medical Record, Established 1870.

Vol. VIII. ATLANTA, GA., JANUARY, 1907. No. 10.

